# Fertility Preserving Management of Refractory Postpartum Haemorrhage: A Case of Bilateral Internal Iliac Artery Ligation Following Uterine Atony

**DOI:** 10.7759/cureus.70908

**Published:** 2024-10-05

**Authors:** Paidi Naga Rachana, Sohana P Budihal, Bharathna Chennuru

**Affiliations:** 1 Obstetrics and Gynaecology, Dr. D. Y. Patil Medical College, Hospital and Research Centre, Dr. D. Y. Patil Vidyapeeth (Deemed to be University), Pune, IND

**Keywords:** fertility preservation, internal iliac artery ligation, obstetric haemorrhage, post partum haemorrhage, uterine atony

## Abstract

Postpartum hemorrhage (PPH) is one of the leading causes of maternal morbidity and mortality worldwide, particularly in low-resource settings. Despite advances in obstetric care, PPH continues to pose significant challenges, especially when conservative management fails. In such cases, more aggressive surgical interventions become necessary to control hemorrhage and preserve the patient's fertility. The bilateral ligation of the anterior division of the internal iliac arteries (IIAL) is a fertility-preserving procedure. This procedure has been widely adopted in obstetric practice, particularly for managing cases of uterine atony, placenta previa, and other conditions associated with massive hemorrhage. However, despite its proven efficacy, IIAL remains underutilized due to the technical expertise required and the limited exposure among obstetricians during training. This study aims to advocate for increased training and awareness to enhance its adoption in clinical practice.

## Introduction

Postpartum hemorrhage (PPH) needs more aggressive surgical interventions. One such intervention is the bilateral ligation of the internal iliac artery (IIAL), a fertility-preserving procedure that has been employed as an alternative to hysterectomy in cases of severe obstetric hemorrhage. This procedure effectively reduces pelvic blood flow, converting the high arterial pressure to a low-pressure system, thereby promoting hemostasis in cases where other measures have failed [1,2].

Internal iliac artery ligation was first described by Kelly in 1894 as a method to control severe pelvic hemorrhage [2,3]. Since then, it has been increasingly adopted in obstetric practice, particularly for managing cases of uterine atony, placenta previa, and other conditions associated with massive hemorrhage [1,2]. The procedure is especially advantageous because it preserves the uterus, thus maintaining the patient’s fertility potential, which is a significant concern for many women of childbearing age [4,5].

Recent studies have demonstrated the efficacy of IIAL in controlling hemorrhage and preventing hysterectomy in obstetric emergencies. For instance, a study by Madhubala et al. highlighted that in a cohort of 31 patients with placenta previa, the implementation of bilateral internal iliac artery ligation significantly reduced the need for postpartum hysterectomy [2]. Similarly, Raba introduced a novel, minimally invasive technique for IIAL that not only reduces the complexity and duration of the procedure but also enhances the learning curve for physicians, making it a more accessible option in emergency settings. This innovative approach addresses the technical challenges often associated with the traditional Kelly’s method, which requires extensive tissue dissection and poses a higher risk of iatrogenic injuries [3].

The success of IIAL in obstetric practice is further corroborated by a retrospective study conducted over 10 years, which involved 11 cases of severe obstetric and pelvic hemorrhage. The study found that IIAL was effective in 10 out of 11 cases, with only one patient requiring an emergency hysterectomy due to the failure of the procedure [6]. This underscores the potential of IIAL as a life-saving measure that preserves fertility while effectively managing hemorrhage.

Moreover, the role of IIAL extends beyond obstetric cases to include severe pelvic hemorrhages resulting from gynecological surgeries. For example, Sivalingam and Rajesvaran reported a rare case of coital injury leading to life-threatening hemorrhage, which was successfully managed with bilateral internal iliac artery ligation [7]. This case further emphasizes the versatility of the procedure in various clinical scenarios, making it a crucial skill in both obstetric and gynecological practice.

Despite its proven efficacy, the adoption of IIAL is not as widespread as expected. This is partly due to the technical challenges associated with the procedure and the lack of adequate training among junior obstetricians and gynecologists [6]. However, with the development of new techniques and increased awareness of its benefits, IIAL is gaining recognition as a valuable alternative to more radical interventions like hysterectomy [5,8]. Notably, studies have shown that IIAL does not adversely affect pelvic organ function or fertility, with patients successfully achieving pregnancies post-procedure [9,10].

To this end, the study aims to highlight the effectiveness of bilateral IIAL as a fertility-preserving intervention in the management of severe PPH, particularly in cases where conventional methods fail. By presenting this case, we seek to underscore the procedure's potential to reduce the need for hysterectomy and improve maternal outcomes, thereby advocating for its broader adoption in obstetric practice.

## Case presentation

The patient is a 35-year-old gravida 3, para 1, with a history of one living child and one abortion. She presented at 35 weeks of gestation with abdominal pain and premature rupture of membranes. Her obstetric history was significant for a previous lower segment cesarean section (LSCS) performed nine years ago due to breech presentation. Additionally, she had a history of uterine leiomyoma, for which a laparoscopic myomectomy was performed on the anterior wall of the uterus seven years ago. During the current pregnancy, she experienced per vaginal (PV) bleeding at two months of amenorrhea, for which progesterone support was administered until 34 weeks of gestation.

Upon admission to the labor room, the patient was found to be vitally stable with uterine size corresponding to 36 weeks and adequate uterine contractions. On per vaginal examination, the cervix was 2 cm dilated, 40% effaced, and the membranes were absent, with the fetus in a breech presentation. Given the previous LSCS and the onset of labor, the decision was made to perform an emergency cesarean section.

During the LSCS, a healthy male infant weighing 2.7 kg was delivered. The placenta and membranes expelled spontaneously bits of membranes that were removed manually, and intravenous oxytocin (20 units) was administered. Despite these measures, the uterus failed to contract adequately, and active bleeding from the endometrial cavity was observed, indicative of uterine atony. Initial medical management of the PPH was attempted by 20 units of oxytocin drip, 800mcg of tab misoprostol sublingual, injection methergine 0.25mg I/m and injection carboprost 250mcg I/m. Injection methergine 0.25mg in three doses and injection carboprost 250mcg in six doses were repeated in total but were unsuccessful in restoring uterine tone.

**Figure 1:**
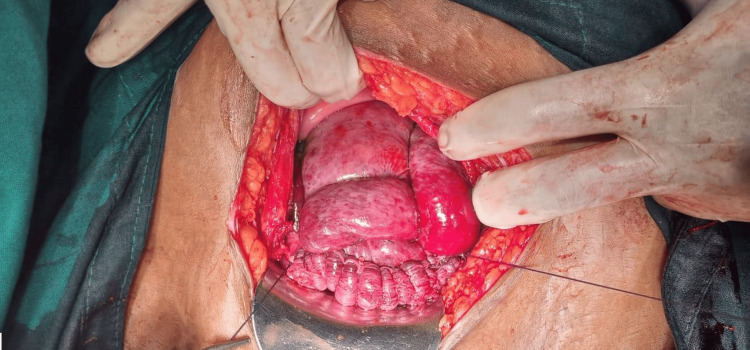
B-Lynch Sutures as a Compression Suture Technique

Given the ongoing hemorrhage, a B-Lynch suture, a compression suture technique, was applied to the uterus to mechanically compress it, as shown in Figure 1.

**Figure 2:**
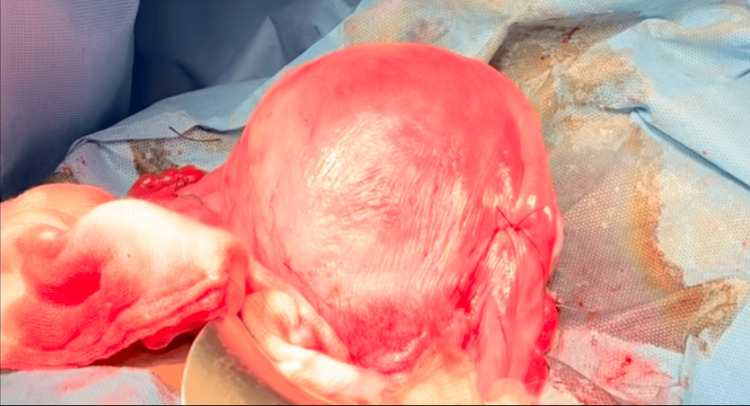
Uterine Artery Ligation by O’Leary Technique

Despite the B-Lynch suture, the uterus remained atonic, necessitating further surgical intervention. Bilateral uterine artery ligation by O’Leary technique was performed to reduce the arterial blood supply to the uterus and control the bleeding, as depicted in Figure 2. However, persistent uterine atony warranted additional measures.

**Figure 3:**
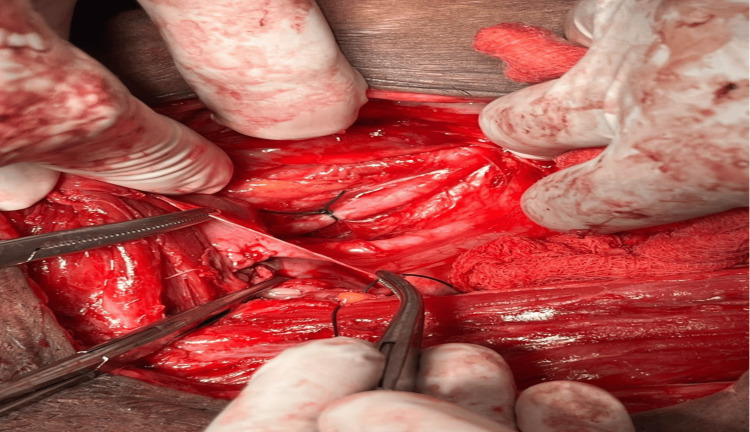
Anterior Division of Internal Iliac Artery (Right Side) 1cm Below the Bifurcation of Common Iliac Artery

**Figure 4:**
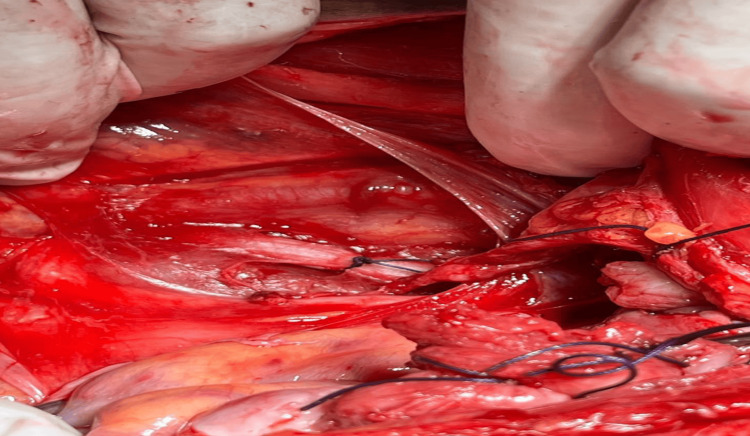
Anterior Division of Internal Iliac Artery (Left Side) 1cm Below the Bifurcation of Common Iliac Artery

Subsequently, the decision was made to perform bilateral ligation of the anterior divisions of the internal iliac arteries, a procedure aimed at significantly reducing pelvic blood flow to control the hemorrhage. The ligation was performed first on the right side, as shown in Figure 3. Anterior division of the internal iliac artery (right side) 1cm below the bifurcation of common iliac artery, and then on the left side, as depicted in Figure 4. Anterior division of the internal iliac artery (left side) 1cm below the bifurcation of common iliac artery as a step of devascularisation procedure. One unit of packed red blood cells was transfused intraoperatively to manage the blood loss.

An intra-abdominal drain was placed before closing the abdomen in layers. The patient was then transferred to the intensive care unit (ICU) for close monitoring and further management. She recovered well postoperatively and was subsequently shifted to the general ward on the third postoperative day. The patient was discharged on the ninth postoperative day in stable condition.

## Discussion

PPH remains a major cause of maternal morbidity and mortality worldwide, accounting for a significant proportion of obstetric emergencies. Bilateral IIAL has emerged as a critical fertility-preserving intervention in such scenarios, offering a surgical option that effectively reduces pelvic blood flow and promotes hemostasis [11]. 

The utility of IIAL in controlling PPH is well-documented. As highlighted by Sarma, IIAL transforms the high-pressure arterial system into a low-pressure venous system, thereby facilitating the formation of clot and stabilizing hemorrhage [12]. The procedure, first described by Kelly in 1894, has since gained prominence in managing obstetric hemorrhages such as uterine atony and placental abnormalities, providing an alternative to hysterectomy, especially in young women desiring future fertility [12]. Despite its efficacy, IIAL remains underutilized due to the technical expertise required and the limited exposure many obstetricians have to the procedure during training [11].

An anatomical understanding of the internal iliac artery and its branches is paramount for the successful execution of IIAL. Anatomical variations, particularly in the branching pattern of the internal iliac artery, can complicate the procedure and increase the risk of intraoperative complications. A study conducted on 50 cadaveric pelvises underscored the variability in the origin, division, and branching of the internal iliac artery, emphasizing the importance of preoperative anatomical assessment and intraoperative vigilance [13]. Awareness of these variations not only aids in avoiding iatrogenic injuries but also enhances the success rate of the procedure by ensuring accurate ligation of the targeted vessels [13].

The clinical benefits of IIAL extend beyond the immediate control of hemorrhage. The procedure has been shown to significantly reduce the need for blood transfusions and lower the risk of hysterectomy in cases of intractable PPH. A study conducted over five years involving 46 patients demonstrated the effectiveness of IIAL in managing PPH and minimizing intraoperative blood loss during gynecological surgeries [14]. The procedure was successful in controlling hemorrhage in the majority of cases, with only one patient requiring subsequent hysterectomy [14]. These findings underscore the role of IIAL as a lifesaving intervention that not only preserves fertility but also reduces the need for blood transfusion, thereby mitigating the risks associated with transfusion-related complications [14].

The successful outcome, with the patient recovering without requiring a hysterectomy, underscores the value of IIAL as part of a comprehensive strategy to control severe PPH. When compared with similar cases in the literature, this case is particularly noteworthy due to the patient's complex obstetric history, including a previous cesarean section and laparoscopic myomectomy. The combination of prior uterine surgeries increased the complexity of managing the PPH and heightened the risk of complications. For instance, in a study by Win et al., a similar approach involving B-Lynch sutures and IIAL was employed in a patient with severe PPH; however, the presence of extensive uterine scarring resulted in more challenging hemostasis [9]. In contrast, the current case demonstrates the successful application of IIAL in a similarly high-risk scenario, further validating the procedure's effectiveness in preserving fertility without additional complications.

The technical execution of IIAL, while demanding, is a crucial determinant of its success. The modified extraperitoneal technique, as described by Sarma, offers several advantages, including reduced operative time, minimal disruption of peritoneal structures, and lower risk of infection [12]. This approach is particularly beneficial in emergency settings where rapid hemostasis is required. However, the success of the procedure hinges on the surgeon's familiarity with pelvic anatomy and the ability to navigate the anatomical variations commonly encountered in the region [12].

In addition to its role in obstetrics, IIAL has been successfully employed in the management of hemorrhages resulting from gynecological malignancies and pelvic trauma. The versatility of the procedure is highlighted by its application in diverse clinical scenarios, ranging from controlling bleeding after hysterectomy to managing intractable hemorrhage during gynecological cancer surgeries [11]. The reduction in blood loss during such procedures not only improves surgical outcomes but also enhances patient recovery by limiting the need for extensive blood transfusions [11].

The discussion surrounding alternative techniques for controlling PPH, such as vaginal uterine artery ligation, further emphasizes the relevance of IIAL. The meticulous dissection required to avoid such complications underscores the advantages of IIAL, where the direct visualization of the target vessels allows for a more controlled and precise intervention [15].

Ultimately, the successful management of PPH through IIAL requires a multidisciplinary approach that integrates surgical expertise with a thorough understanding of pelvic anatomy and hemodynamics. The evidence from various studies reinforces the procedure's efficacy, not only in preserving fertility but also in ensuring maternal survival in life-threatening situations [16,17]. The continued refinement of techniques and the broader adoption of IIAL in obstetric practice could significantly reduce the incidence of hysterectomy and improve outcomes for women experiencing severe PPH.

This discussion emphasizes the critical role of bilateral IIAL as a lifesaving, fertility-preserving intervention in severe PPH cases that are unresponsive to conventional treatments. The successful outcome in this case underscores the importance of IIAL in a multimodal approach to managing refractory PPH, advocating for enhanced surgical training and broader adoption of this technique in obstetric care to improve maternal outcomes and preserve reproductive potential.

## Conclusions

PPH remains a leading cause of maternal morbidity and mortality. Bilateral IIAL is a proven and effective intervention in controlling severe obstetric hemorrhage when other conservative measures fail. This fertility-preserving procedure serves as a critical alternative to hysterectomy, particularly for young women who desire future pregnancies. The success of IIAL lies in its ability to reduce pelvic blood flow and transform the high-pressure arterial system into a low-pressure venous system, thereby promoting hemostasis. Despite its proven efficacy, the widespread adoption of IIAL is hindered by the technical expertise required and the anatomical variations that complicate the procedure.

The case presented demonstrates the practical application of IIAL in a 35-year-old patient with severe PPH due to uterine atony following a cesarean section. The combination of B-Lynch sutures, uterine artery ligation, and IIAL ultimately led to the successful control of hemorrhage without necessitating a hysterectomy. This outcome highlights the importance of a multi-modal approach to managing refractory PPH, where IIAL plays a pivotal role.

Looking forward, it is essential to incorporate IIAL training into obstetric residency programs to ensure that more practitioners are skilled in this life-saving procedure. Additionally, ongoing research into improving surgical techniques and understanding the anatomical variations of the internal iliac artery will further enhance the success rates of IIAL. As obstetric care continues to advance, IIAL should remain a cornerstone in the management of severe PPH, ensuring better maternal outcomes and preserving reproductive potential.
